# Investigation of Removal Capacities of Biofilters for Airborne Viable Micro-Organisms

**DOI:** 10.3390/ijerph15030551

**Published:** 2018-03-19

**Authors:** Rémi Soret, Jean-Louis Fanlo, Luc Malhautier, Philippe Geiger, Sandrine Bayle

**Affiliations:** 1 LGEI, IMT Mines Ales, University of Montpellier, 30100 Ales, France; remi.soret@mines-ales.fr (R.S.); jean-louis.fanlo@mines-ales.fr (J.-L.F.); luc.malhautier@mines-ales.fr (L.M.); 2 CMI Europe-Environnement, 1 rue des Pins, Parc d’Activités du Pays de Thann, 68700 Aspach-Michelbach, France; philippe.geiger@cmigroupe.com

**Keywords:** biofiltration, bioaerosols, BioTrak, fluorescence, operating parameters

## Abstract

New emerging issues appears regarding the possible aerosolization of micro-organisms from biofilters to the ambient air. Traditional bioaerosol sampling and cultural methods used in literature offer relative efficiencies. In this study, a new method revolving around a particle counter capable of detecting total and viable particles in real time was used. This counter (BioTrak 9510-BD) uses laser-induced fluorescence (LIF) technology to determine the biological nature of the particle. The concentration of viable particles was measured on two semi-industrial pilot scale biofilters in order to estimate the Removal Efficiency in viable particles (RE_vp_) in stable conditions and to examine the influence of pollutant feeding and relative humidification of the gaseous effluent on the RE_vp_. The RE_vp_ of biofilters reached near 80% and highlighted both the stability of that removal and the statistical equivalence between two identical biofilters. Pollutant deprivation periods of 12 h, 48 h and 30 days were shown to have no influence on the biofilters’ removal capacity, demonstrating the robustness and adaptation capacities of the flora. In contrast, a 90-day famine period turned the biofilters into emitters of viable particles. Finally, the humidification of the effluent was shown to negatively influence the removal capacity for viable particles, as drying off the air was shown to increase the RE_vp_ from 60 to 85%.

## 1. Introduction

Air pollution control has been a concern for many years. To preserve air quality and limit emissions of chemical pollutants, several laws have been established at national, European and international scales. For example, the regulations toward air pollution control tightened over the last years for Volatile Organic Compounds (VOCs) and odors (Directive 2008/50/EC). To comply with those regulations, biological processes such as biofilters have been implemented for the treatment of gaseous effluents in a wide range of environments like manufacturing industries (paper industries, chemical industries, etc.), wastewater treatment plants, biogas production units (methanization) or composting plants [1]. Biofilters are one of the best available techniques [1,2]. They are appropriate for treating gaseous stream with high flow rate at low concentration of pollutants. They offer a sustainable and eco-friendly solution for air pollution control, with low maintenance costs. For all those reasons, their implementation at industrial scale grew exponentially in recent decades. However, as biofilters are biological systems themselves, further focus has been set on the question of if they act as additional sources of airborne microorganisms. Hence, the dispersion of microorganisms from gas biofilters and their health consequences for workers and residents are the subject of discussions. During the biofiltration process, gaseous effluent is forced through a packing bed material colonized by a microbial flora which enables the degradation of the pollutant [3].

This biological mechanism leads to interrogations about the possible aerosolization of micro-organisms from the biofilm to the gaseous effluent and their diffusion in the atmosphere [4,5,6]. Some studies demonstrate adverse health effects from occupational exposure to bioaerosols produced during biological process functioning [7]. These questions have led to the publication of Directive 2000/54/EC by the European Parliament on the protection of workers from risks related to exposure to biological agents at work. The British Government recently emitted a new regulation enforcing the monitoring of bioaerosols in biological waste treatment plants [8]. In ambient air, the World Health Organization (WHO) indicated that the exposure limit value (ELV) of bioaerosol in ambient air should be lower than 300 CFU·m^−3^ [9].

Moreover, the inlet gaseous influent can contain a large amount of micro-organisms or some pathogenic micro-organisms according to the industrial activity (rendering, biogas production …). A few studies investigated those questions by using generally classical microbiological methods [6]. However, it is well known that these methods are rather laborious (depending on the culturability of microorganisms) and time consuming (from 2 to 10 days) [10]. Moreover, Amann et al. [11] reported that the percentage of cultivable micro-organisms in an environment represent only from 0.01 to 10% of the total microbial flora. Cultivable cells only represent 4 to 30% of the total biomass in gas biofilters [12,13]. Furthermore, the collection methods and growth media used to study emissions of micro-organisms from gas biofilters were variable.

The profusion of approaches has made it difficult to compare results [6,9,14,15,16,17,18,19,20]. To overcome these problems, new approaches called rapid microbiological methods (RMM) appeared. Among them, methods to determine the number of microorganisms via evaluation of microbial viability have been developed [21]. One of those emerging techniques is based on laser/light-induced fluorescence (LIF) which is used by commercially available devices such as the BioTrak^®^ counter. The counter’s utilization has been validated by the USFDA [22] and its performances as an alternative method of microbiology have been validated in accordance with USP Validation of Alternative Microbial Methods (USP <1223> [23], USP <1116> [24]), the European Pharmacopoeia (Ph. Eur. Chapter 5.1.6 [25]) and the Parenteral Drug Association (PDA Technical Report 33 [26]). This device allows an easy and fast monitoring of total viable airborne flora with a cut-off size of 2 μm [27], which is relevant to the 2–10 μm aerodynamic diameter range where the majority of bioaerosol can be found [28]. In their review, Fletcher et al. [6] highlighted the need for regular monitoring of bioaerosol emissions from biofilters during steady state operation by using a standardized and repeatable measurement method. Moreover, it is also of great importance to examine the influence of operating parameters on the potential emission of microorganisms.

They also emphasized the use of a parameters controlled biofiltration pilot unit to eliminate the high variability of industrial field sampling. The BioTrak counter could be an efficient tool for that purpose. The first goal of this study is then to determine the RE_vp_ of a biofiltration pilot unit at steady state by monitoring its emissions of viable particles. The second goal is to investigate the influence of operating parameters on the RE_vp_ via controlled perturbations. The influence of two operating parameters such as pollutant feeding and humidity of the effluent has been presented here.

## 2. Materials and Methods

### 2.1. Optical Total and Viable Particles Counter BioTrak^®^

The main device used in this study is the Optical Total and Viable Particles Counter BioTrak^®^ (BioTrak 9510-BD, TSI, Shoreview, MN, USA). The counter is composed of two distinct stages. The first one hosts an Optical Particles Counter (OPC). This counter uses laser diffraction to measure and count every solid particle, including inorganic and organic particles, in the diameter range from 0.5 to 25 μm. Among these particles, viable particles are then determined by auto fluorescent detection.

For this detection, the particles go through a cyclonic concentrator which reduces the airflow while maintaining the majority of the particles inside, to then reach the second stage.

This corresponds to the viability detector which uses laser-induced fluorescence to discriminate the viable particles in the total exiting the concentrator. For this, two 405 nm wave length lasers enable the fluorescence of the NADH, Flavin and tryptophan molecules associated with viability.

This device allows the real-time monitoring of the viable airborne flora. The sampling flowrate is 28.3 L·min^−1^. Measurements using the BioTrak counter consisted of 15 cycles: a measurement of 1 min followed by a pause of 30 s.

### 2.2. Pilot Scale Biofiltration Unit

**Figure 1:**
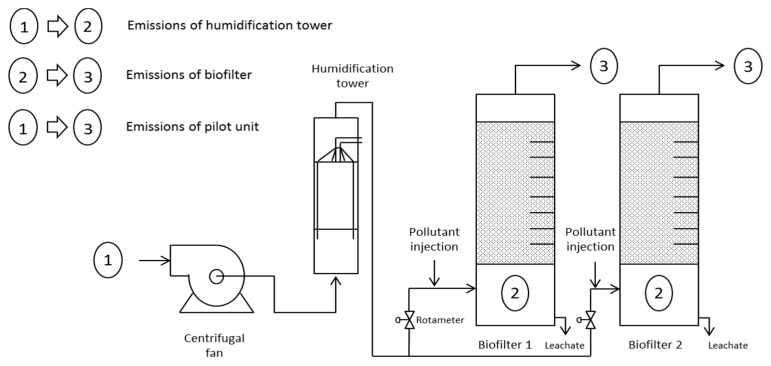
Schema diagram of experimental design with particles sampling points. 1: Ambient air. 2: Gas homogenization area. 3: Gas outlet.

The unit is composed of 2 major parts: the gaseous effluent generation and the two biofiltration units in parallel (Figure 1).

A synthetic gaseous effluent containing a carbon concentration of around 7.5 g·carbon·m^−3^ air at a flow rate of 20 L·min^−1^ was created by injecting SP95 petrol into compressed air stream prior to reaching the carrier effluent. The carrier effluent is composed of ambient air aspired by a centrifugal air fan and going through a humidification tower to reach maximum water saturation (95%) before being distributed among the biofilters, in an upward flow mode, at a flow rate of 2 m^−3^·air·h^−1^ and an inlet VOCs concentration of 1 g·carbon·m^−3^. Biofiltration columns are identical PVC columns of 3 m in height and 30 cm in diameter and packed with sawmilled wooden chips with an average size of 10 mm up to 1 m height. Both were operated in parallel during all experiments. A 50 cm head space at the top of each column contained the humidification system. A 55 cm bottom section is designed for gas velocity homogenization prior to entering the packing bed, as well as for leachate recovery. In order to maintain the humidity of the packing bed material, the biofilters were watered with standard water at a flow of 15 L·m^−2^·day^−1^.

The biofilters were inoculated by placing the packing bed material inside sewage sludge coming from the local wastewater treatment plant for 24 h. Ambient temperature was controlled in the laboratory test hall assuring a minimum temperature of 15 °C. The inlet stream load was of 360 g of pollutant per cubic meter of packing bed per day^−1^ (360 g·m^−3^·day^−1^).

For this study, the supply of pollutant followed an alternation of 12 h of feeding followed by 12 h of deprivency for 5 days (Monday to Friday), then a period of complete deprivation for 2 days (Saturday and Sunday). This set up was chosen to represent typical full-scale biofilters operation in the oil industry (refining, storage, treatment of petroleum wastes). The average pollutant removal efficiency during this study was of 30%.

### 2.3. Experimental Design

#### 2.3.1. Sampling Points

A schematic representation of the sampling points used is presented in Figure 1.

The first sampling point corresponded to the ambient air. It was placed approximately at 1.5 m from the floor and 1.5 m from the fan aspiration to ensure isolation and representativeness of the samplings. The second sampling location was inside the gas homogenization area and was sampled through the leachate outlet.

The third sampling point corresponds to the air exiting the biofilter, and was sampled directly inside the head space at the top of the column within a space located at 10 cm from the packing material. The RE_vp_ of the pilot unit as a whole, of the humidification tower, and of the biofilter could be estimated by comparing the sampling results of points 1 and 3; points 1 and 2; points 2 and 3 respectively.

The results are expressed as removal efficiency of viable particles (RE_vp_). The RE_vp_ is calculated as follows:

(1) $$\mathrm{RE}\;\mathrm{vp}\;\left(\%\right)=\left(\frac{C\;\mathrm{input}-C\;\mathrm{output}}{C\;\mathrm{input}}\right)\times 100$$

where C input corresponds to ambient air concentration (VP·m^−3^) and C output to outlet gas concentration (VP·m^−3^).

#### 2.3.2. RE_vp_ at Steady State

The emissions of viable particles of biofilters at steady state were investigated and the RE_vp_ determined during 44 days for both columns operating with strictly identical conditions. Two measurements per day were conducted to take into account both daily variations and pollutant feeding alternation. The first measurement was performed in the morning and the second one in the afternoon.

#### 2.3.3. Pollutant Feeding

The influence of pollutant feeding was studied by depriving the biofilters of any pollutant for controlled periods of time while maintaining the same airflow. The first starvation period represented the alternation of pollutant feeding at steady state, thus deprivation periods of 12 and 48 h. The second starvation period was of 90 days and started after a 3 months period of steady state. Following this starvation period, the biofilters were put back in steady state operations for 15 days. The final starvation period took place afterward and lasted 30 days. During feast conditions, both biofilters were fed with basal VOCs concentration (360 mg·m^−3^). Each famine period was only applied when all biofilters experienced steady state.

#### 2.3.4. Gas Humidification/Moistening

The influence of the air moisture content was examined by shutting down the humidification tower of the pilot unit for up to 50 h. No further time was added in order to preserve the biofilm on the packing bed material.

A complementary analysis of the quantity of water in the air was performed during this period. Both air temperature and relative humidity were determined by using a temperature and hygrometry probe (KIMO instruments, Montpon Ménestérol, France), and the quantity of airborne water was computed with data from a Mollier diagram. Measurements were carried out in the ambient air, at the bottom and at the top of the biofilter to estimate the emitted quantity of water for each section of the experimental design by using a water mass balance.

The determination of viable particle quantities in the air using the BioTrak counter was simultaneously performed, thus allowing the specific isolation of the share of the biofilter itself in the removal efficiency of the total pilot unit.

### 2.4. Statistical Analysis

Different statistical tests were used during this study. Analysis of variance was used in priority. For non-normally distributed variables, data were compared with Wilcoxon test. ANOVA was used to analyze steady state and humidification studies’ data. Wilcoxon test was used for the pollutant deprivation study. The equivalence one-sided test (TOST) was used to determine the statistical equivalence between both biofilter columns. All analyses were performed using the statistic software R. The chosen level of risk was 5%, a result was thus considered statistically significant with a *p*-value < 0.05.

## 3. Results and Discussion

### 3.1. Steady State

Viable particles’ concentrations (VP concentration) were monitored at steady state for a period of 44 days for both biofilters. The variation of the inlet VP concentration in the entire study (steady state, influence of pollutant and influence of humidity included) was limited to around 1 log maximum, ranging from 900 to 12,000 VP·m^−3^. The median inlet concentration was calculated as being 3200 VP·m^−3^ and the mean concentration was 3800 ± 2400 VP·m^−3^ which highlighted the stability of the concentration throughout the study. The outlet VP concentration during the steady state study ranged from 270 to 3700 VP·m^−3^ with a mean concentration of 950 ± 750 VP·m^−3^ for BF1. The concentration ranged from 20 to 2600 with a mean concentration of 970 ± 750 VP·m^−3^ for BF2. On average, the emissions were also lower than what reported in the literature [5,29,30,31,32,33,34,35,36,37]. However, the majority of these studies revolved around field sampling on on-site biofilters in highly contaminated environments. However, these results are higher than the WHO recommendation for bioaerosol concentration (300 UFC·m^−3^) [9].

**Figure 2:**
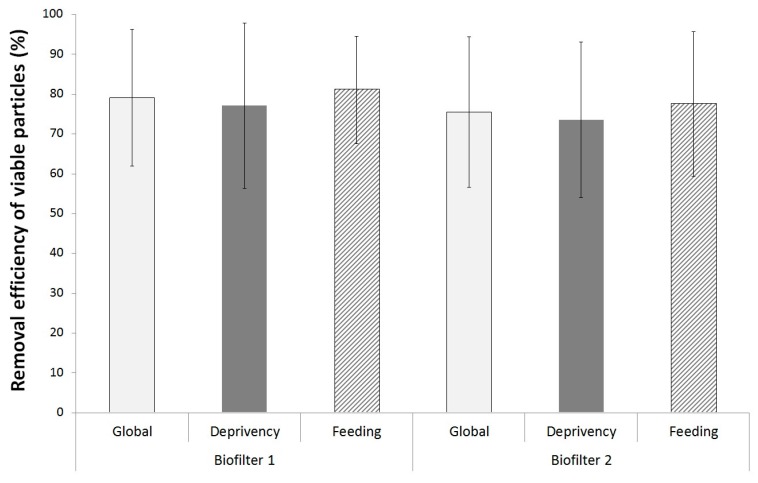
RE_vp_ for both biofilters during the pollutant alternation of deprivency and feeding periods (*n*_deprivency_ = 25/bars, *n*_feeding_ = 27/bars).

The small variation in VP concentrations in inlet effluent ensured the representativeness of the RE range variations. This parameter was therefore chosen to monitor changes in biofilter removal capacities of VP. RE_vp_ values are presented in Figure 2. The RE_vp_ were of 79 ± 17% and 76 ± 19% for biofilter 1 (BF1) and biofilter 2 (BF2) respectively.

These values were in agreement with microbial RE values which have been reported by different authors for bacterial and fungal communities: from 30–98% for bacteria [5,29,30,31,32,33,34,35,36,37], and from 49–100% for total fungi [5,29,30,31,32,33,37]. It is important to underline that these previous works were performed using on-site industrial biofilters. Hence, design characteristics, operating parameters and packing bed materials of these biofilters differed from one another. Furthermore, results were gathered using different sampling devices and sampling methods. The observed seemingly high variability among all these data is then due to both biofilter design and operation, and microbial particles sampling and counting. It should also to be noted that those results (except for [35]) were obtained through culturable methods with significant delays. It is also well known that these methods involve challenges for the representation of airborne microorganisms [10]. According to Amann et al. [11], these studies could have overlooked up to 90% of the total airborne microbial flora. The BioTrak technology has been chosen to overcome the cultivability of micro-organisms and to improve the microflora representation.

A few studies have implanted culture independent methods to observe airborne micro-organisms from biofilter. Epifluorescence Microscopy and FISH analysis have been used by Esquivel-Gonzalez et al., 2017 [9] and Ho et al., 2008 [35] respectively. Esquivel-Gonzalez et al., 2017 have found a weak emission from their biofilters (average inlet concentration: 3 × 10^7^ cells·m^−3^ air and average outlet concentration: 3.8 × 10^7^ cells·m^−3^·air). Using the FISH technique, HO et al., 2008 found a RE for total micro-organisms of 90% and 98% which is concordant with our results.

Epifluorescence Microscopy include in their quantification non-viable cells or microbial fragments in the count (Esquivel-Gonzalez et al., 2017). Comparison of the target to be measured could explain the variation in the results. Culture methods highlight viable and cultivable micro-organisms population, BioTrak targets the viable micro-organisms, EM detects total cells (viable, dead and fragments) and FISH detects the DNA. There is a need to explore further the differences in measurement of different technologies.

Statistical analysis showed no difference between the feeding and deprivency periods (*p* = 0.3). Both 12 and 48 h of deprivency period included in the normal operation feeding scheme had no effect upon the RE_vp_ levels emitted by biofilters (Figure 2). Moreover, no statistical difference has been detected on RE_vp_ between both biofilters (*p* > 0.8).

The equivalence test demonstrated that both biofilters, identical in term of designs and operating conditions, presented equivalent removal efficiency for viable particles (*p* < 0.05).

### 3.2. Pollutant Feeding

Viable particles concentrations have been monitored to test the ecosystem resistance to a famine period. Both biofilters have been submitted to different famine periods (12 h/48 h, 30 days, and 90 days).

**Figure 3:**
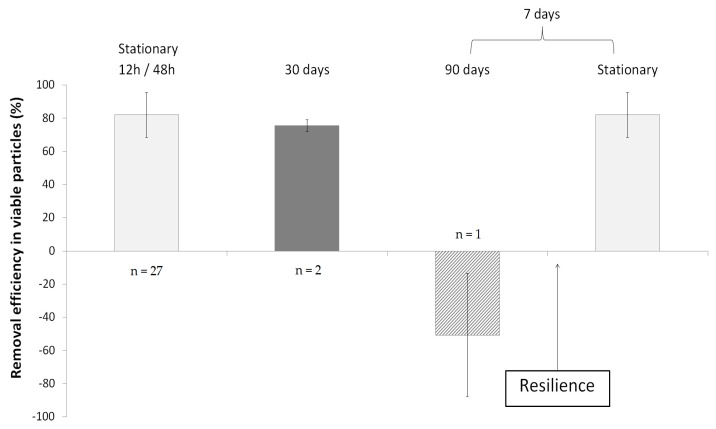
RE_vp_ during the different deprivency periods (12 h/48 h, 30 and 90 days). The time required to recover the RE_vp_ at steady state is of seven days.

The RE_vp_ have been calculated and summarized in Figure 3. In stable operating conditions (12 h/48 h famine periods) identical efficacy of 79 ± 17% have been observed (Section 3.1). Two deprivation periods of 30 days have been performed, the mean outlet VP concentration was 960 ± 700 VP·m^−3^. The average RE_vp_ was of 76 ± 4%.

During the 30 days period, the outlet concentration was remarkably similar to the steady state ones, showing no modification of the RE_vp_. During the 90 days deprivation period, the mean inlet concentration was 2700 ± 1500 VP·m^−3^. The data showed a negative average removal efficiency of −51 ± 37% during this period. The outlet concentration was significantly higher and highlighted the microbial aerosolization. After the seven day recovery period, the average concentration went back to 1000 ± 640 VP·m^−3^ and ranged from 570 to 1500 VP·m^−3^. The concentration after the seven day recovery period also underscored the resilience of the system as it went back to steady state equivalent level.

None of the 12 h, 48 h and 30 days deprivation periods had an influence on the levels of the RE_vp_ (*p* < 0.01). Biofilter turned into an emitter of viable particles with a negative average RE_vp_ (*p* < 0.001) after 90 days of pollutant deprivation. When both biofilters were re-supplied with pollutants, a single period of seven days was required to reverse the phenomenon back to steady state, with an average efficiency level similar to that obtained before the 90-day famine period.

Those results highlighted several phenomena.

The robustness of biofiltration toward short-term famine, (12–48 h and 30-day periods) could supposedly come from the ability of the microbial flora to use the substrate as a source of energy, which is an already reported phenomenon [38,39,40,41,42]. When the concentration of pollutant severely dropped during the deprivation period, the biofilter’s microflora was able to slow down its metabolism and triggered a famine adaptation mechanism. It is probable that both carbon and energy sources were supplied by the packing material to compensate for the lack of pollutant.

Then, the microbial community organization was not disturbed for at least 30 days, as seen here, with no modification on the level of viable particles within the aerosol and the RE_vp_.

However, after a longer famine period (90 days) microbial emission was observed, as around 50 ± 37% more micro-organisms exited the biofilter than entered it. All RE_vp_ values remained under 0% thus further highlighting the aerosolization of viable particles. This period seemed to be sufficient to overcome the adaptation mechanism of the microbial community. As the environmental conditions for microbial growth were unfavorable, the microflora developed a strategy of conservation (migration strategy) to find another suitable environment.

When both biofilters were re-supplied with a polluted gaseous effluent, a rapid return (one week) to the steady state RE_vp_ was observed. The pollutant removal efficiency also took one week to return to pre-disturbance level (data not shown). This highlighted the resilience of biofilters toward long term famine. The possible explanation is that the renewed access to food stopped the migration survival mechanism of the microbial flora.

The results seemed to highlight the robustness of biofilters in terms of viable particles removal and that the biofilters and the associated microflora could survive long-term famine periods (at least 30 days).

### 3.3. Influence of the Effluent Humidification

The measurements at the foot and the top of the biofilter allowed for the specific isolation of the biofilter column and showed that it possessed an average removal efficiency of 90 ± 4%. During the test period with the humidification tower working, the inlet concentration was of 2900 ± 1500 VP·m^−3^ (*n* = 6), while the inlet concentration was of 2400 ± 400 VP·m^−3^ (*n* = 3) without humidification. The levels of the inlet concentrations were thus similar in both cases. As seen in the steady state study, this difference in the concentrations range could not explain the drastic difference between the RE_vp_ with air humidification and the RE_vp_ without humidification. The difference in the RE_vp_ thus could not be imputed to a drastic difference of the inlet concentration but to the removal of air moisture. In the same way, the mean outlet VP concentration were of 1200 ± 420 VP·m^−3^ during humidification and 300 ± 160 VP·m^−3^ without, which highlighted the augmentation of the RE_vp_ due to air moisture removal. In addition, these results demonstrate that the biofilter is the main part of the RE_vp_, thus confirming the biofilter ability to remove viable particles.

The RE_vp_ levels have been obtained with a pilot unit containing a humidification tower before the biofilter (Figure 1). To further investigate the particles retention of the biofilter itself, the determination of particle concentration between the tower and biofilter was attempted. However, the air humidity in this area prohibited the use of counter. Consequently, the humidification tower was stopped for up to 50 h which allowed to investigate of the biofilter RE_vp_ itself.

When the humidification tower was working, the quantity of water delivered to the gas was estimated at 18 g·h^−1^ Table 1a. At the same time, the humidity content in the gas at both inlet and outlet of the column was similar which emphasized the stability of the moisture of the packing bed through time.

The quantity of water at the outlet of the biofilter itself has been determined after the humidification tower has been stopped (Table 1b). The obtained values seem to highlight the drying of the packing bed material (the quantity of water at the biofilter outlet was of 22.5 g·h^−1^ and of 15 g·h^−1^ at 22 and 46 h respectively).

**Figure 4:**
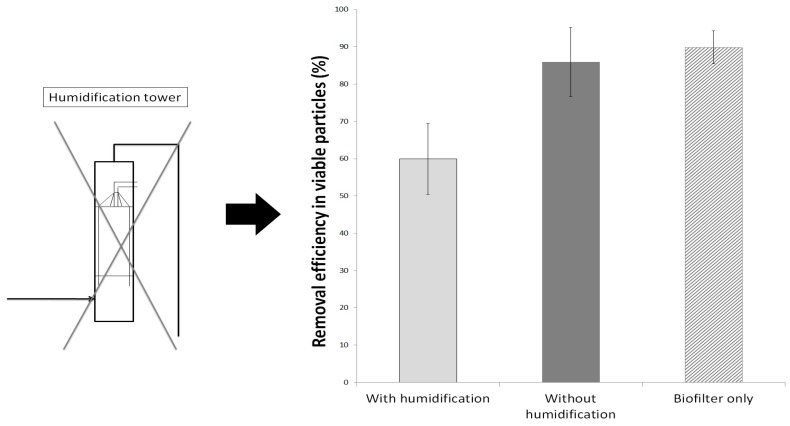
RE_vp_ during normal operation humidification and after 46 h without humidification. “With humidification” and “Without humidification” correspond to the evaluation of viable particles counts by using one and three sampling points. “Biofilter only” corresponds to the evaluation of viable particles counts by using sampling points 2 and 3 (*n* = 2).

The levels of RE_vp_ of the pilot unit with or without humidification as well as the RE_vp_ of biofilter itself are presented in Figure 4. Those results exhibited an average RE_vp_ of 60 ± 9% during normal operation with humidification for the pilot unit in totality. After 46 h without air moistening, the average RE_vp_ increased to an average of 86 ± 9% which is statistically different to when the gaseous effluent is moistened (*p* < 0.03).

When the packing bed material dried, the RE_vp_ increased drastically. The absence of water saturation of the packing bed seemed to decrease the crossing of the biofilter by the microbial flora, or its aerosolization.

Vergara-Fernandez et al., 2012 [43] have reported an opposite result: a decrease of fungal spores’ concentration concomitant with an increase of packing material. This opposite result could be explained by several factors. Actually, the scale up effect of the biofilter (1000 L for us against 0.5 L) must be taken into account, as the scale-up effects inherent to a 2000% augmentation in volume can drastically change the results. Biofilter used in this study is packed with a consortium while a mono specie biofilter was used by Vergara-Fernandez et al., 2012, which directly affected the diversity of the bioaerosols emitted. Sporulation effects had a major influence on the results. The humidity increase may have altered the sporulation. Finally, the method of detection could have influence the result. Here, culture independent method is used. Further studies are necessary on the influence of humidity in order to get a clear picture.

This phenomenon could be explained as follows. The first assumption could be linked to the loss of cell viability when the air humidity ratio is reduced, thus resulting in a higher death rate among the microbial flora and the increase of the RE_vp_.

However, the same concentration of viable particles was found at the fan aspiration and at the bottom of the biofilter when the air humidification was stopped, hence demonstrating a RE_vp_ of 0% in the humidification tower. These results lead to think another explicative phenomenon is possible.

**Figure 5:**
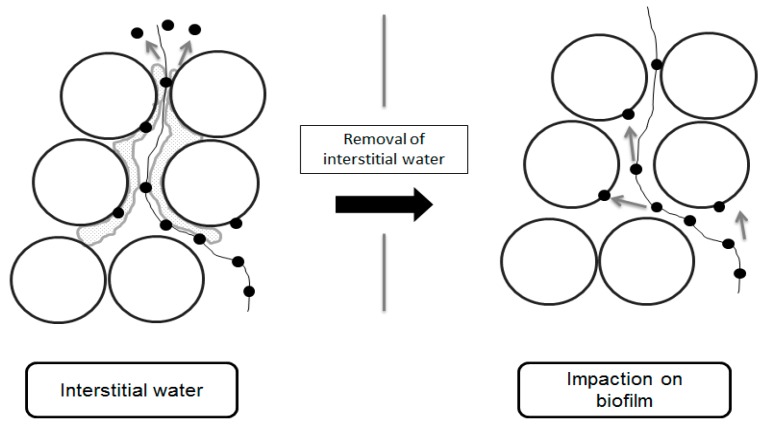
Explaining mechanism: disappearance of interstitial water and enhancement of the impaction phenomenon.

This second phenomenon could be due to the increase of the impaction phenomenon when the interstitial channels dry out (Figure 5). In normal water-saturated operation, the gaseous effluent travels through the packing bed material and passes near or through the interstitial water, creating turbulences, bubbles and agitation in the water. The impaction of micro-organisms into a collection liquid has been studied through the impingement technology, including the re-aerosolization mechanisms [20,44]. Even inside specifically designed collection devices, a consequent amount of liquid-impacted micro-organisms are re-aerosolized due to the effects of the airflow on the water. It is then possible that the interstitial water act as a collection liquid when the air passes near, with a consequent amount of micro-organisms re-aerosolized from it which then reached the outlet effluent.

When the relative humidity of air is reduced, the passage of water-unsaturated air stream through a water saturated packing bed induces the volatilization of water molecules from interstitial water to restore liquid gas equilibrium. Consequently, the biofilm becomes thinner. The diffuse water phase diminishes, and the water level comes closer to the poly-saccharides structures of the biofilm [45,46,47,48,49]. The micro-organisms have easier access to the complex microscopic surface of the wooden chips. The re-aerosolization phenomenon is then reduced inducing the augmentation of the RE_vp_.

The relative humidity of both gaseous effluent and packing bed are then important parameters to ensure pollutant degradation and airborne viable particles removal capacities of biofilters.

Moreover, these results highlight the opposition between the best conditions for viable particles removal and for pollutant removal, as already highlighted by Fletcher et al. [6]. However, further tests are needed to confirm these results by exploring the composition of microbial communities notably using biomolecular tools.

## 4. Conclusions

This study brings new insight on viable particle emissions by biofilters. It has been highlighted that biofilters act as filters for viable micro-organisms. With stable conditions, a high and stable RE_vp_ was maintained around 77% for both biofilters and enlightened the statistical equivalence between the two identical biofilters. Furthermore, this effect was linked to the biofilter itself and not to the humidification tower. The robustness of biofilters has been emphasized: a long pollutant deprivation period was required to largely reduce the viable particle RE_vp_, thus showing the resistance and resilience of the microbial flora toward short term famine. Moreover, it has been stressed that interstitial water can negatively affect the RE_vp_. Consequently, it was demonstrated that the operating parameters can influence the RE_vp_.

Moreover, these results highlighted how the best operating conditions for airborne viable particles control are opposed to the best pollutant degradation conditions. This could indicate that if viable particles removal also becomes a focus, a compromise and balance should be reached in order to maximize both the pollutant and the RE_vp_ but with the inability to reach the highest possible level for each.

## Figures and Tables

**Table 1:** (**a**) Air temperature and relative humidity at the different localizations in the pilot unit, airborne water concentration calculated using Mollier diagram and airborne water flow corresponding to the airflow (2 m^3^·h^−1^). (**b**) Quantity of water exiting the biofilter through time after the tower stoppage, calculated using airborne water mass balance between the bottom and the top of the biofilter.

**(a)**	**T (°C)**	**HR (%)**	**C_eau_ (g_eau_·kg_air_^−1^)**	**Q_eau_ (g·h^−1^)**
Ambiant air	20	55	8	19
Tower exit	21.5	95	15	37
Biofilter exit	21.5	95	15	37
**(b)**	**Time**		**Biofilter**	
	3 h		22 g·h^−1^	
	22 h		23 g·h^−1^	
	46 h		15 g·h^−1^	
